# Generating realistic retinal image for whole visual system modeling

**DOI:** 10.1186/1471-2202-12-S1-P348

**Published:** 2011-07-18

**Authors:** Takayuki Kannon, Shiro Usui

**Affiliations:** 1 RIKEN Brain Science Institute, Wako, Saitama, 351-0198, Japan

## 

Recent progress and improvements on optical technology have enabled us to measure the characteristics of the eye in great detail. It has also been shown that, even when myopia and astigmatism are completely corrected with spectacle, the retinal image is still blurred due to uneven refraction power of the cornea and the crystalline lens [1][2], an effect known as “irregular astigmatism”. However, almost all researches in visual science assume sharp images as input.

Currently, a large-scale whole visual system models is being developed on a supercomputer to elucidate its complex function [3]. Moreover, it has been demonstrated that the blurred retinal images are gradually compensated at both, the retina and the cortex [4]. Therefore, we have developed an eye optics model which can calculate the blurred retinal image based on known physiological evidences to provide a more realistic input to retinal and visual cortex model.

The proposed model is described by the linear image filter based on Artal's model [5]. It can process multi-spectral image for reproducing the spectral characteristics of photoreceptors. OTF (Optical transfer function) calculated from the wavefront aberration with spectral transmittance of the lens is used for image processing filter. The wavefront aberration is defined by Zernike Coefficients values (measured by wavefront aberrometer such as OPD-Scan or Shack-Hartman Sensor systems) or SCA values (power of sphero-cylindrical lens with its axis for spectacle lens or contact lens prescriptions). To consider the effects of diffraction, OTF is designed to vary depending on pupil diameter and wavelength. To account for aging effects, the spectral transmittance of the lens [6] was also implemented. As inputs of the model, multispectral images taken by multispectral camera or converted from RGB image can be utilized.

We simulated a closed-loop system connecting the proposed model to a simple eye movement model and pupil model. The eye movement model generates random saccades and the pupil model modifies pupil diameter according to the mean luminance of retinal image. As a result, our model could reproduce the effect of chromatic aberration, pupil diameter and age on the blurred retinal image. By applying our model to the large-scale visual system model, it is expected to be able to evaluate the phenomena, for example, age dependent perception on visual illusions, blur compensation in retina or visual cortex, and so on.
